# Unmasking speed curve anomalies in team sports: a practical guideline for data treatment and interpretation

**DOI:** 10.3389/fspor.2026.1750588

**Published:** 2026-02-20

**Authors:** Rui Marcelino, Hugo Silva

**Affiliations:** 1 Research Center in Sports Sciences, Health Sciences and Human Development, CIDESD, ELITE Research Community, Maia, Portugal; 2 University of Maia, Maia, Portugal; 3 FPF Academy, Portuguese Football Federation, Oeiras, Portugal

**Keywords:** acceleration, athlete-tracking, deceleration, maximum speed, sprint

## Abstract

Monitoring high-speed displacements in team sports commonly relies on maximal values, often referred to as Peak Match Speed (PMS). These values are widely used to guide training prescription, injury-prevention strategies, and performance profiling. However, PMS metrics may be distorted by anomalous events, such as tackles or collisions, which generate implausible speed–time patterns and compromise the accuracy of player monitoring. The purpose of this commentary is to present a practical strategy to identify and resolve such abnormalities, thereby increasing the reliability of athlete-monitoring processes. Systematically plotting acceleration-time and speed-time curves together, with the acceleration axis aligned to PMS, allows practitioners to rapidly detect unrealistic patterns, such as extreme accelerations or decelerations near maximal speeds, that deviate from physiological expectations. By identifying and excluding these artefacts, practitioners ensure that derived metrics more accurately reflect players' true physical capacities. This proposed strategy is adaptable to a wide range of team sports and can also enhance the interpretation of submaximal sprint efforts. Importantly, this low-cost and widely applicable approach strengthens the reliability of athlete-tracking outputs, safeguarding both performance analysis and training decision-making.

## Introduction

The monitoring of players in team sports has become a widely studied area, with technological advances providing increasingly precise and relevant insights for practitioners. While early monitoring relied on simple indicators such as session duration or total distance, current approaches allow detailed characterization of high-intensity efforts, with particular emphasis on high-speed displacements given their association with performance, fatigue, and injury risk (1–3). Within this context, maximum running speeds have received particular attention, as these values not only provide an indication of physical potential but also serve as a reference point for individualized training prescription and load monitoring (4–6). Here, the term Peak Speed denotes the highest values observed, in line with previous research across various sporting contexts (7, 8). Moreover, Peak Match Speed (PMS) refers specifically to peaks attained in competition to distinguish them from field-based test values (9).

The use of PMS therefore reflects both the practical constraints of current monitoring systems and the applied relevance of such measures in team sport settings. Increasingly, while field tests can distinguish faster from slower players (10), they are not replicable during competition (11). Consequently, practitioners increasingly rely on peak speeds from training and competition, systematically tracked and updated through modern global navigation satellite system technologies whenever higher values are reached. Although PMS is derived from match play, it is widely used in applied practice as a reference value to prescribe and individualize training, ensuring that players are adequately prepared for the physical demands of competition. PMS thus emerges as a highly relevant indicator for both training and competition, serving not only as a benchmark of physical capacity but also as a reference in injury prevention strategies (12). Importantly, peak speeds comparable to, or even exceeding, PMS can be (and often should be) achieved during training, depending on session design and objectives. Yet no consensus exists on where maximum speed is most likely to be expressed – testing, training, or competition – and this may vary with sport-specific demands (13, 14).

With global navigation satellite systems (GNSS), speeds are usually calculated through the Doppler shift, by measuring the change in frequency of the satellite signal caused by the movement of the receiver (15). Then, speeds can be exported as raw data and treated according to the practitioners' objectives. However, this data includes noise (such as speeds irrelevant fluctuations) which leads manufactures to apply filters. These filters, such as minimum effort duration criteria (e.g., considering only efforts lasting longer than 0.5 s) (16), typically aim to attenuate high-frequency noise and signal artefacts. While providing a meaningful help to practitioners, manufacturer-filtered data may rely on predefined or threshold-based approaches, which have been shown to present limitations in reliability and sensitivity (17). Alternatively, raw data enables deeper analysis in comparison with filtering data that mostly rely on what effort has happened rather than how it happened. Hence, access to raw data allows practitioners and researchers to assess signal quality at a finer temporal resolution, apply individualized thresholds, and retain information on rapid changes in speed that may otherwise be smoothed or excluded by filtering processes. A clear example of this limitation is the possibility of evaluating match peak speed curves (18), providing an important information regarding how players accelerate to and decelerate from high-speed displacements. Additionally, practitioners can evaluate signal quality (e.g., satellite connection and horizontal dilution of precision) during specific moments, rather than relying on a single session-level average, which may be misleading (19).

Hence, to use peak speeds as reliable reference values, practitioners must ensure measurements are accurate and error-free. Although rare, a single error can compromise data quality: if an erroneous maximal effort is accepted, all derived values (speed, acceleration, deceleration) risk distortion, leaving the player with misleading “maximums” for an entire season. Deeper analysis is therefore needed to understand the circumstances of such efforts. Examining the full speed-time curve can reveal movement patterns and highlight abnormalities. This commentary illustrates how speed-time analysis can expose events that interfere with high-speed metrics while offering a simple, practical solution.

Raw Global Navigation Satellite System (GNSS) data were collected using a multi-GNSS augmented unit (STATSports Apex®, Newry, Northern Ireland), operating at 10 Hz sampling frequency. Prior to data collection, all devices were activated 30 min before match play to ensure optimal satellite signal reception, with a minimum constellation of eight satellites confirmed for each observation period. This technical approach aligns with recommendations from recent research using similar GNSS technologies in professional football contexts and ensures full replicability of the analytical workflow. Raw data were exported containing latitude, longitude, and timestamp information. Velocity and acceleration were subsequently calculated using custom-developed Python routines (authored by the lead researcher), which processed the raw positional coordinates and temporal information. All derived metrics, including speed-time curves and acceleration-time patterns, were calculated from these processed raw data without relying on manufacturer-applied filters at the analysis stage. This approach enabled fine-grained temporal resolution (0.1-s intervals) and allowed practitioners to assess signal quality at specific moments rather than relying on session-level averages. The raw export approach retained information on rapid changes in speed that might otherwise be smoothed or excluded by proprietary filtering processes, thereby enhancing the ability to detect anomalous patterns within individual sprint efforts.

## A smooth speed-curve – the practitioners’ expectation

**Figure 1:**
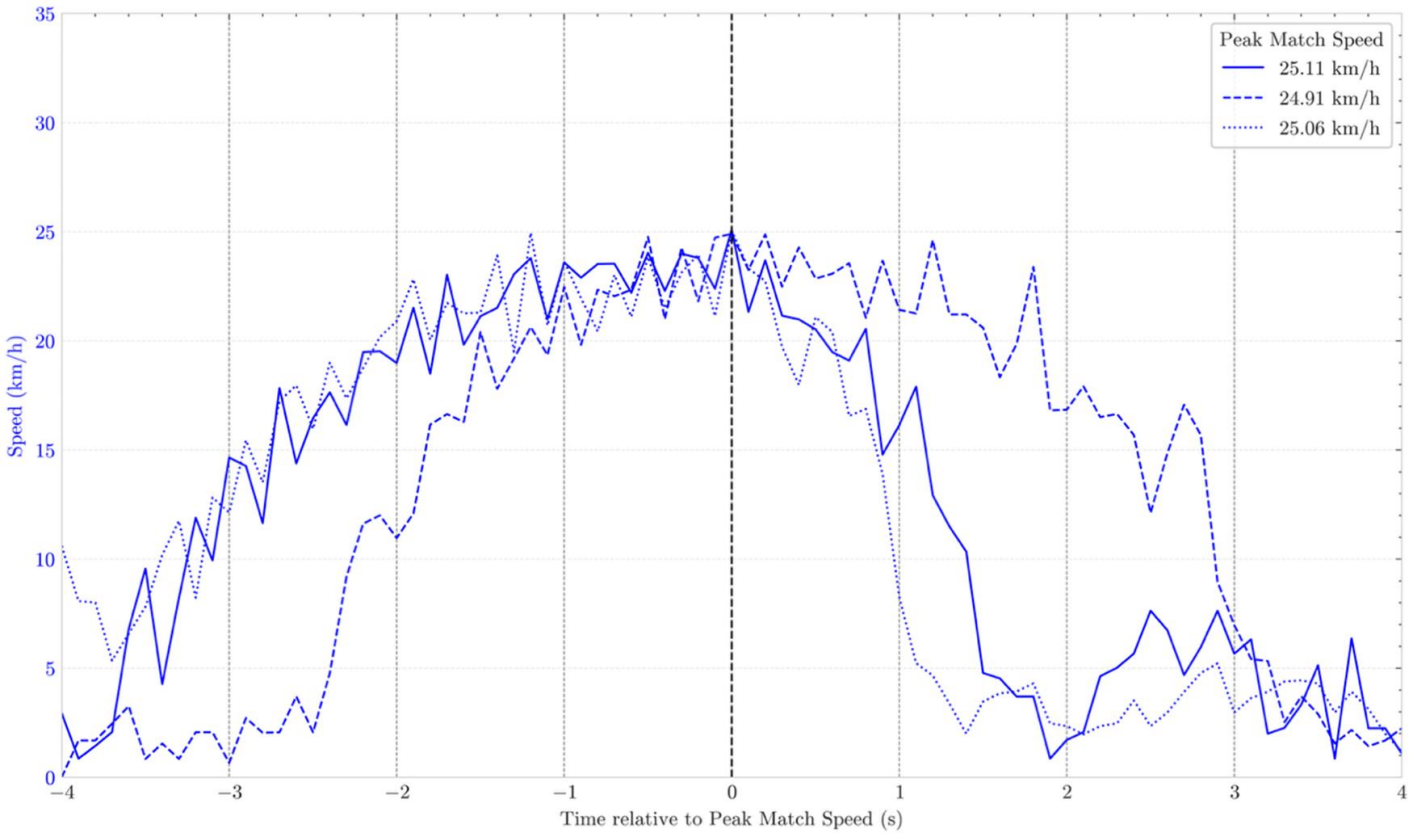
Speed–time curves of three PMS efforts performed by the same player in three separate competitive matches. Curves are centered on the moment of PMS (time = 0 s), with data shown from 4 s before to 4 s after the peak. Small fluctuations are visible and reflect either rapid changes in acceleration or the temporal resolution of data collection.

Upon collecting performance data, practitioners can isolate specific occurrences for deeper analysis, allowing each high-speed displacement to be examined individually. Although these efforts can be synchronized with video, the data alone may already reveal meaningful patterns. Figure 1 illustrates three speed curves from independent efforts during a beach soccer match. As expected, speed rises progressively to PMS before declining more rapidly – though not abruptly – a pattern also reported in soccer (18). Of note, while some irregularities may appear in the curve, small fluctuations are normal due to speed being recorded at 0.1-s intervals. For the sake of clarity and coherence, all examples presented in this commentary derive from the same player, monitored during a single competitive season, and exclusively from match data, with no training information included.

Small fluctuations along the speed curve are to be expected and do not necessarily compromise the interpretation of the overall effort, as they often reflect rapid changes in acceleration or the temporal resolution of the data collection system. Consequently, although the curve may appear irregular rather than perfectly smooth, such variations are inherent to the natural dynamics of movement and should be distinguished from meaningful deviations that could indicate abnormal occurrences.

## A peak within the peak speed curve – the improbable acceleration and an unrealistic deceleration

**Figure 2:**
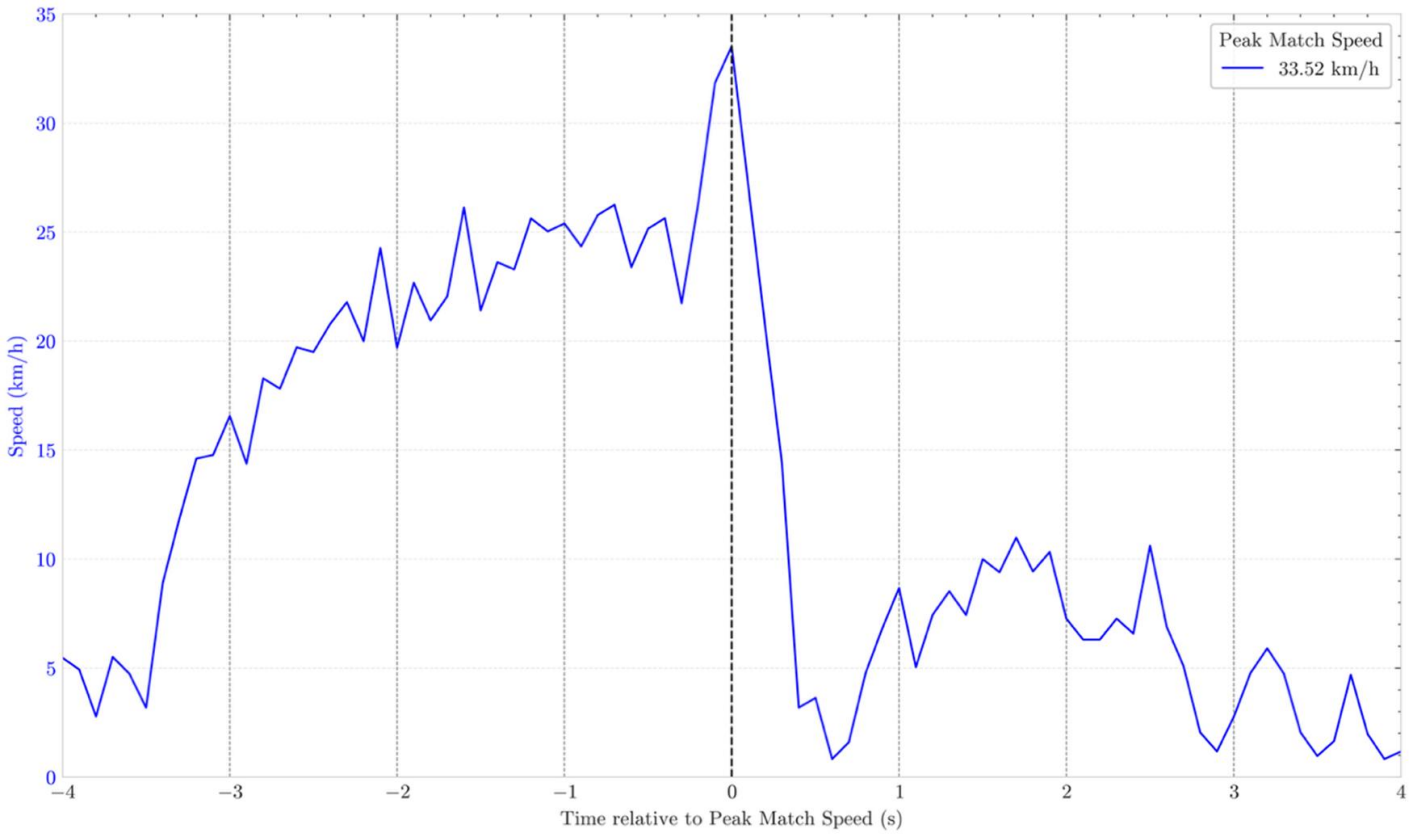
Example of an anomalous speed–time curve observed in a competitive beach soccer match. Besides reporting a new PMS for this player, the curve exhibits a sudden, unusually high acceleration immediately before the PMS, followed by an extreme deceleration. Such patterns are physiologically implausible and indicate the need for further analysis.

While Figure 1 provides important insights into typical PMS efforts, Figure 2 highlights a potential atypical effort. For a player with an approximate PMS of 25 km/h, achieving ∼34 km/h as a new peak value is unexpected. Such an increase should automatically prompt a deeper analysis by practitioners. Moreover, in the three “normal” PMS (Figure 1), acceleration gradually diminishes as speed approaches its maximum, reflecting the expected physiological behavior of running: the capacity to accelerate decreases as speed increases (20, 21). In contrast, the speed-curve in Figure 2 shows a sudden rise immediately before PMS, indicating an acceleration of unusually high magnitude – particularly given the elevated speed at which it begins. Immediately after PMS, a sharp decline in speed further departs from the expected patterns illustrated in Figure 1. Nevertheless, classifying this peak effort as erroneous requires supporting evidence, best achieved by jointly analyzing speed and acceleration.

## Identification, interpretation and resolution of unusual match occurrences

**Figure 3:**
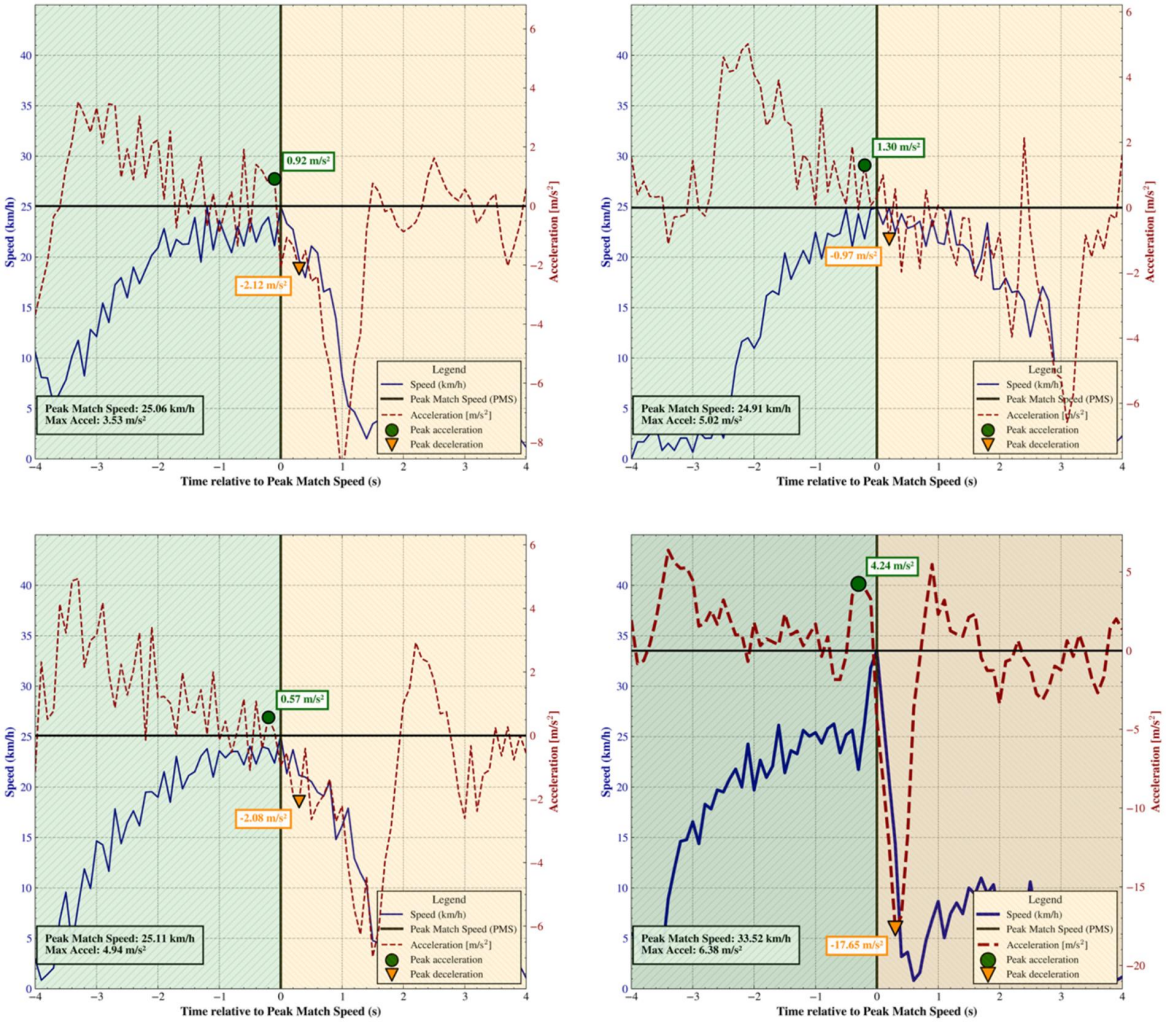
Speed-time and acceleration-time curves from four sprints by the same player during one season. Curves are aligned so that peak match speed (PMS, vertical black dashed line at time = 0) coincides with zero acceleration. In the three typical sprints (top and lower-left panels), acceleration gradually declines toward PMS (marked by green circles). In contrast, the anomalous sprint (lower-right, shaded) shows unrealistically high acceleration (4.24 m/s^2^, marked by green circle) before the peak and extreme deceleration (−17.65 m/s^2^, marked by orange inverted triangle) after PMS, illustrating how combined curves help identify abnormal maximal-speed events. Bold annotations indicate the peak acceleration and deceleration values for each sprint.

Figure 3 presents four panels combining the speed curves shown in Figures 1, 2, with the lower-right panel (shaded background) illustrating the potential erroneous speed curve. Here, each curve is aligned such that the moment of PMS coincides with zero acceleration. This alignment is physiologically justified: when an athlete reaches their maximal speed, acceleration must necessarily pass through zero. Such a visualization facilitates the rapid identification of anomalous patterns that might not be apparent when examining speed alone.

As shown in Figure 3, this effort corresponds to an unusually high acceleration just prior to PMS (4.24 m/s^2^), followed almost instantaneously by an extreme deceleration (–17.65 m/s^2^). This pattern is physiologically implausible and indicates an abnormal event, consistent with the irregularities previously highlighted in Figure 2. Accounting for the dependence of acceleration on starting speed, this value is much higher than the maximal magnitudes reported for starting speeds of ∼21.7 km/h (<3 m/s^2^) (20). This indicates that, near maximal speeds, players are unable to produce accelerations of very high magnitude. Moreover, maximal accelerations tend to be lower on sand than on grass (22), which further exacerbates this improbability.

Together, referencing the new PMS and incorporating the acceleration data into the speed curve allows practitioners to identify an anomalous peak-speed event that does not represent the player's true physical capacity and should therefore be excluded from the player database. Importantly, aligning the acceleration axis with PMS greatly enhances the detection of such anomalies: while an acceleration of 4.24 m/s^2^ is not unusual in itself, a peak of this magnitude occurring immediately before PMS is physiologically implausible and clearly signals an artefact. This is evident in final panel of Figure 3.

While video review can clarify unusual events, it may not always reveal unrealistic efforts. In this case, video confirmed that the player had been tackled and propelled onto the sand, explaining the irregularities in the speed and acceleration curves. Such situations are common in contact sports (23, 24) and may pass untagged during match analysis. Because video is not always available or synchronized with tracking systems, practitioners need accessible tools to identify abnormal values. Plotting acceleration alongside speed, with the axis aligned to PMS, enables rapid detection of improbable accelerations or decelerations near peak effort that might otherwise bypass automated filters. Consequently, an inaccurate estimation of PMS, whether due to measurement error in peak detection or the use of arbitrary data-processing methods (25), can have meaningful consequences for training prescription and the evaluation of competitive demands, potentially misinforming both season-long loading strategies and the interpretation of match intensities.

Importantly, potential errors on raw data can also be identified through the availability of satellites and the horizontal dilution of precision (HDOP). While a minimum of four satellites connection has been referred (26), research usually report higher number of satellites (>10), as this can also positively impact the dilution of precision (27). Briefly, HDOP represents the quantification of the distribution of satellites, and its value should ideally be inferior to 1 (a HDOP of 1 would represent that one satellite is directly positioned above the receiver and the remaining satellites are equally distributed across the horizon. However, this does not exclude all potential errors as shown here. For instance, the number of satellites and the HDOP registered in the four speed-curves analyzed here were always >15 and <0.4, respectively. Therefore, all the MPS here discussed could be included as a new value, even the erroneous case. Although illustrated with beach soccer, the approach is transferable across team sports and useful for analyzing both maximal and submaximal sprints. Additionally, it should be acknowledged that this analysis is based on observations from a single player and, therefore, does not imply that such measurement or processing-related issues will occur consistently or across all athletes, as illustrated in Figure 1; nevertheless, any applied analysis should begin by ensuring that all input values are accurate and appropriately verified. Ultimately, distinguishing genuine maximal performances from artefacts is essential for reliable load monitoring and accurate performance evaluation, with future advances in filtering algorithms further supporting practitioners.

## Practical applications

The accuracy of athlete tracking, particularly in monitoring maximal and near-maximal running speeds, is critical for athlete profiling, training prescription, and injury prevention. However, anomalous values arising from collisions, artefacts, or sensor errors may go unnoticed if only single maximal numbers are considered. While an erroneous peak speed value can be automatically excluded with manufacturer's filtering techniques, the use of filtered data unable practitioners from deeper data explorations, which apport meaningful relevance for practice. For instance, by analysing speed-curves, practitioners can prepare training drills to better replicate match demands. This commentary highlights the practical value of routinely analyzing speed–time and acceleration–time curves together. This enables practitioners to verify not only the magnitude of new peaks but also whether the associated acceleration pattern is physiologically plausible at the attained speed. The approach allows suspicious events to be detected even without video support, is applicable across team sports where contact or technical errors distort data and can be replicated for other high-speed efforts.

## Conclusion

This commentary outlines a simple yet effective strategy to interpret maximal speed and acceleration metrics in team sports. Detecting unusual updates in match peak speed and plotting acceleration- and speed-time curves – aligned at peak match speed – helps practitioners distinguish genuine performances from anomalies caused by collisions or tracking errors. This approach is transferable across a wide range of team sports and competitive contexts, including environments with limited or no video support, as it relies primarily on GNSS-derived speed and acceleration data rather than sport-specific observational inputs. Ultimately, this strategy helps practitioners ensure the reliability of high-speed displacement data and supports more accurate athlete tracking.

## Data Availability

The datasets presented in this article are not readily available because Data is private information and is not available for the general public. Requests to access the datasets should be directed to rmarcelino@umaia.pt.
